# Supplementary material for: Regionally sourced bioaerosols drive high-temperature ice nucleating particles in the Arctic
Source: Nat Commun. 2023 Sep 28;14:5997. doi: 10.1038/s41467-023-41696-7 (PMC10539358; doi:10.1038/s41467-023-41696-7)
Supplement: Supplementary file 1 — Supplementary Information [file 41467_2023_41696_MOESM1_ESM.pdf]

# Supporting information: Regionally sourced bioaerosols drive high-temperature ice nucleating particles in the Arctic

**Gabriel Pereira Freitas**[1,2], **Kouji Adachi**[3], **Franz Conen**[4], **Dominic Heslin-Rees**[1,2], **Radovan Krejci**[1,2], **Yutaka Tobo**[5,6], **Karl Espen Yttri**[7], **and Paul Zieger**[1,2,*]

[1]Department of Environmental Science, Stockholm University, Stockholm, Sweden
[2]Bolin Centre for Climate Research, Stockolm University, Stockholm, Sweden
[3]Department of Atmosphere, Ocean, and Earth System Modeling Research, Meteorological Research Institute, Tsukuba, Japan
[4]Department of Environmental Sciences, University of Basel, Switzerland
[5]National Institute of Polar Research, Tachikawa, Japan
[6]Department of Polar Science, School of Multidisciplinary Sciences, The Graduate University for Advanced Studies, SOKENDAI, Tachikawa, Japan
[7]NILU - Norwegian Institute for Air Research, Kjeller, Norway
[*]Correspondence: paul.zieger@aces.su.se

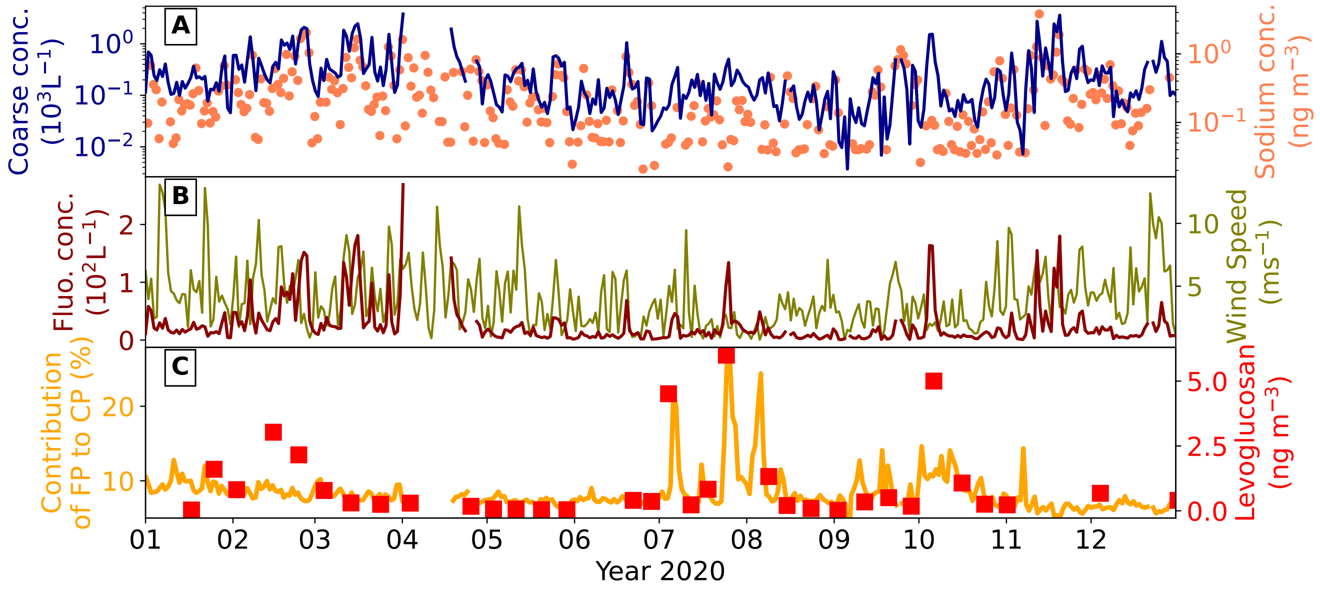


**Figure S1. Timelines of parameters measured during the year 2020 of the NASCENT campaign.** a) Coarse mode concentration as measured by the multiparameter bioaerosol spectrometer (MBS) and sodium concentration in filter samples. b) Fluorescent particle (Fluo) concentration and wind speed. c) Contribution of fluorescent particles (FP) to coarse particles (CP) and levoglucosan particulate concentration.

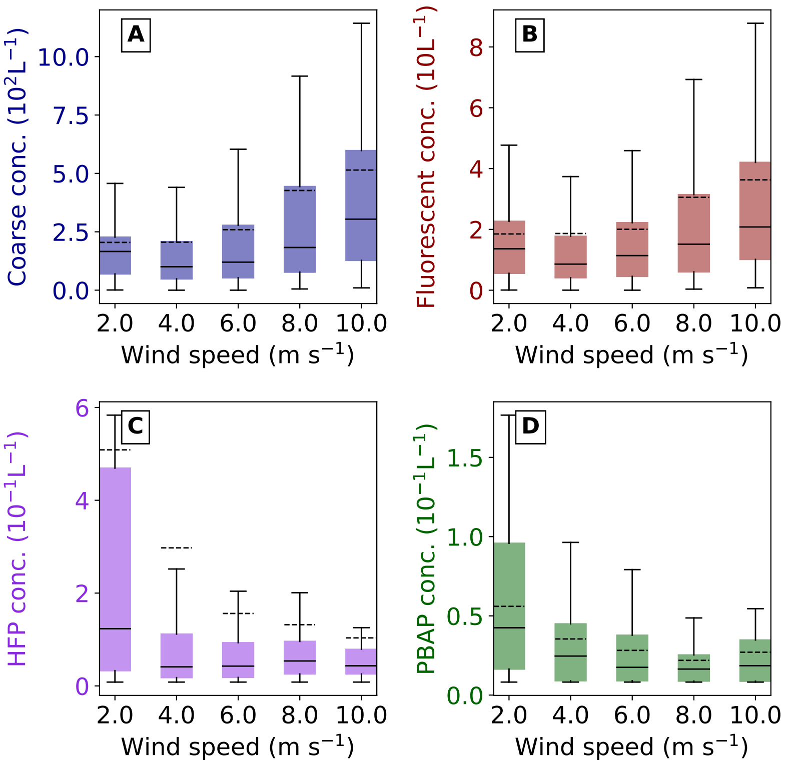


**Figure S2.** **Wind-speed dependency of the main particle classes measured by the multiparameter bioaerosol spectrometer (MBS).** This is shown here for (a) coarse particles, (b) fluorescent particles , (c) highly fluorescent particles (HFP), and (d) primary biological aerosol particles (PBAP). For the boxplots, the continuous line represents the median and the dashed line the mean, the extent of the colored box shows the interquartile range and the whiskers the data range (1.5 times the interquartile range from the nearest quartile).

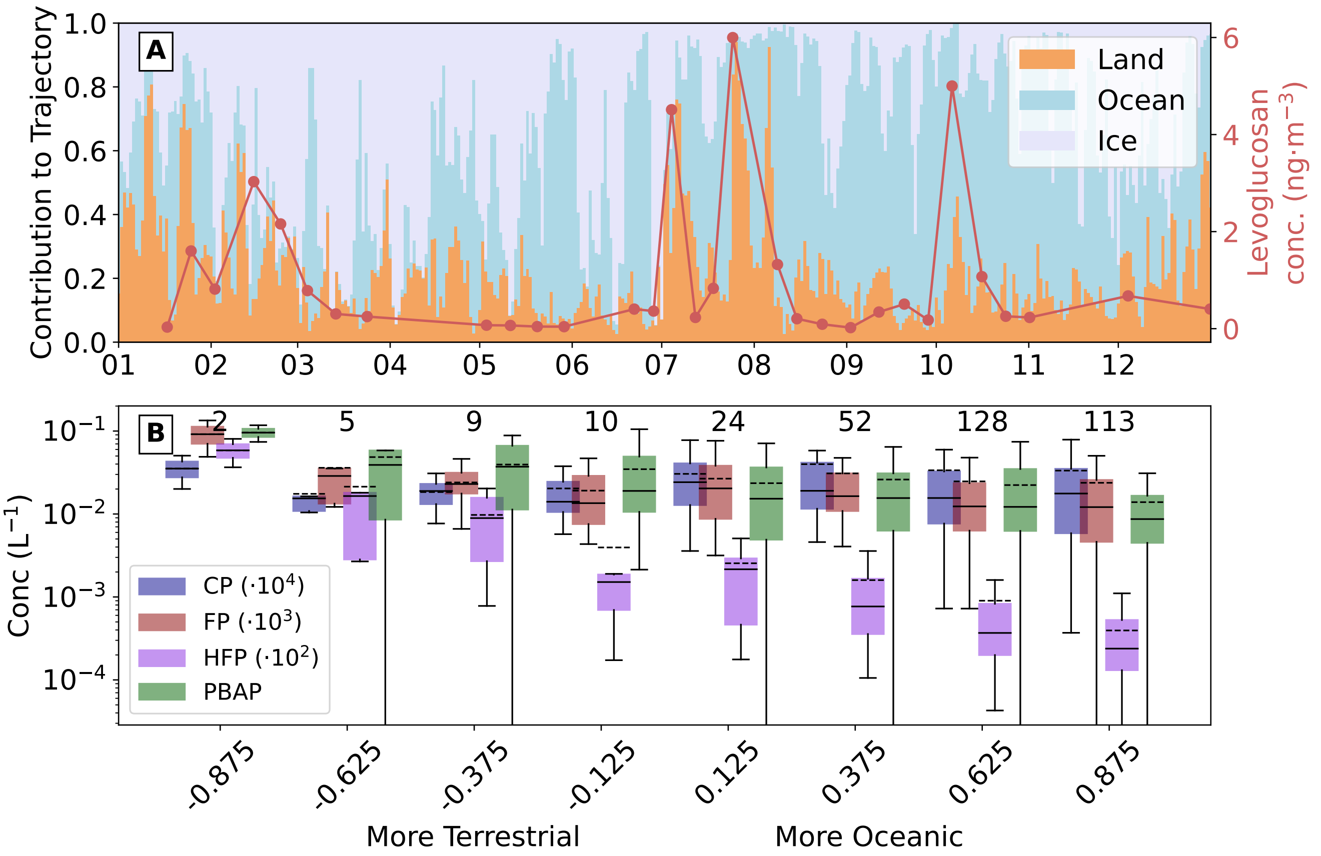


**Figure S3.** **Back trajectory analysis.** (a) Daily back-trajectory contribution over land, ocean and ice superimposed by levoglucosan (red dotted line) filter measurements. (b) Mean concentrations of coarse particles (CP), fluorescent particles (FP), highly fluorescent particles (HFP) and primary biological aerosol particles (PBAP) measured by the multiparameter bioaerosol spectrometer (MBS) categorized for more terrestrial or more oceanic (ocean+ice) back trajectories. The numbers of points per classification are given at the top of the panel. For the boxplots, the continuous line represents the median and the dashed line the mean, the extent of the colored box shows the interquartile range and the whiskers the data range (1.5 times the interquartile range from the nearest quartile).

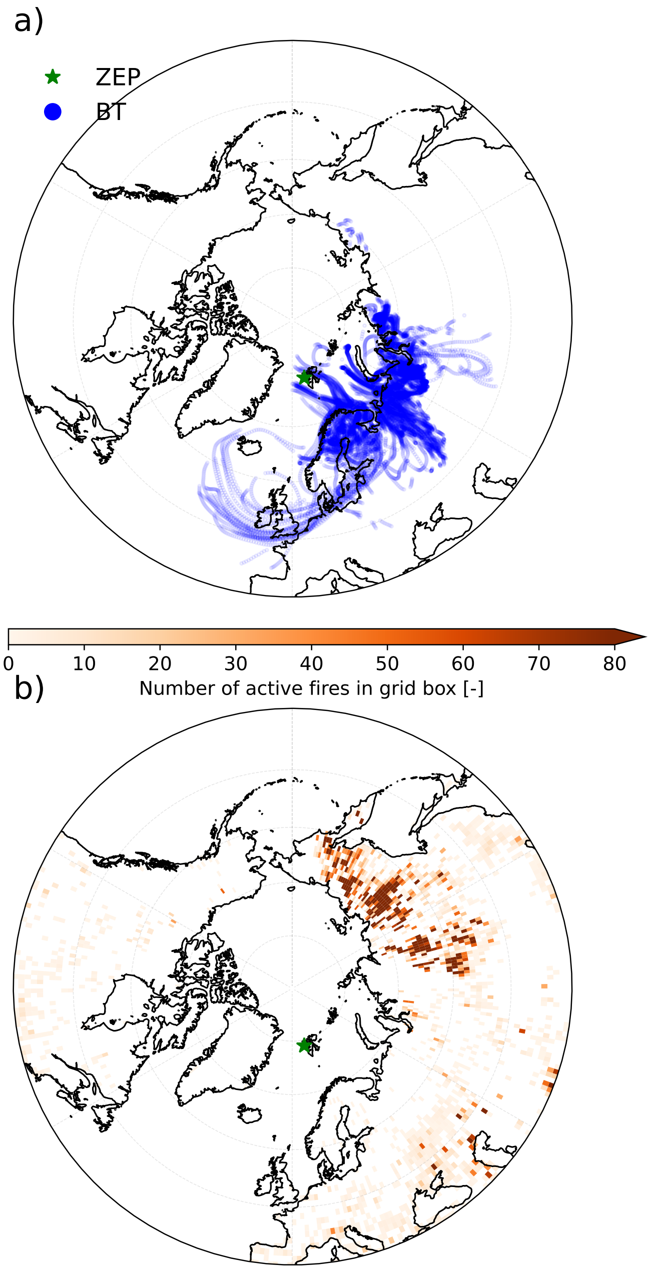


**Figure S4. Back trajectory calculations cross-referenced with active fires for 7 of July of 2020.** (a) 10-days back trajectories (BT) with end point at Zeppelin Observatory (ZEP), where only mixing-layer (ML) was considered. Trajectory points whose altitude sits below the mixing height output by the model are considered to be within the ML. (b) MODIS active fire data was gridded and counted for each BT. Ensemble of trajectories pass through 12597 fires.

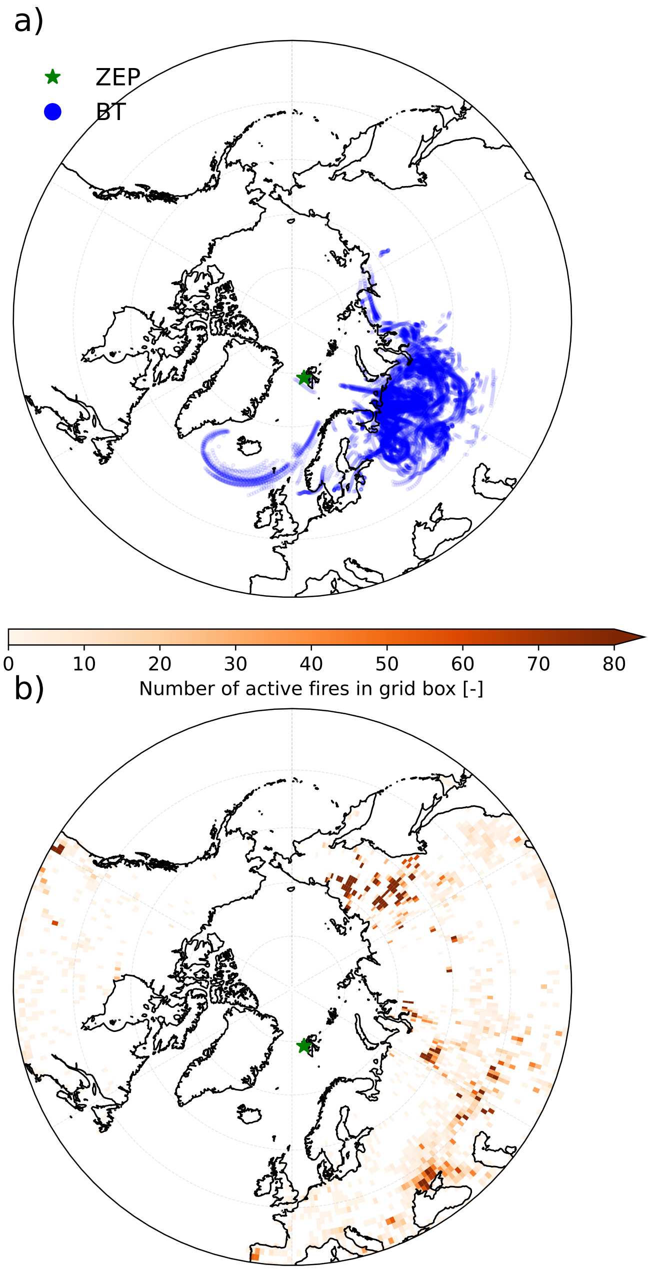


**Figure S5. Back trajectory calculations cross-referenced with active fires for 26 of July of 2020.** (a) 10-days back trajectories (BT) with end point at Zeppelin Observatory (ZEP), where only mixing-layer (ML) was considered. Trajectory points whose altitude sits below the mixing height output by the model are considered to be within the ML. (b) MODIS active fire data was gridded and counted for each BT. Ensemble of trajectories pass through 77091 active fires.

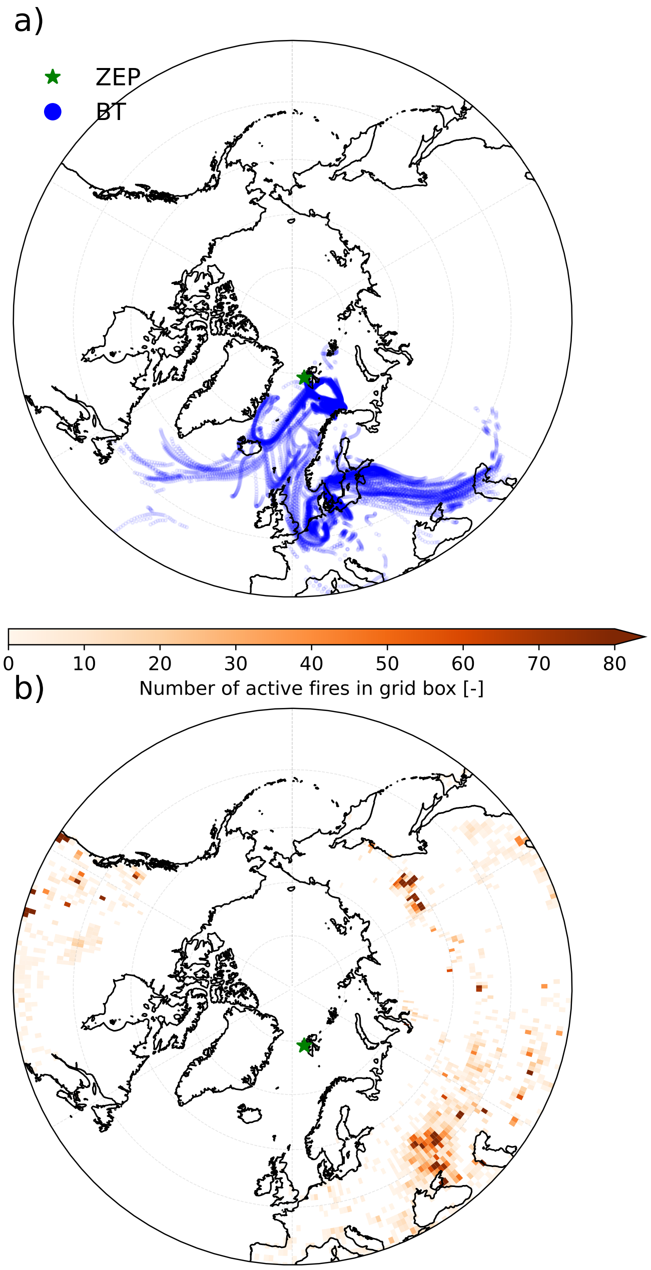


**Figure S6.** **Back trajectory calculations cross-referenced with active fires for 6 of October of 2020.** (a) 10-days back trajectories (BT) with end point at Zeppelin Observatory (ZEP), where only mixing-layer (ML) was considered. Trajectory points whose altitude sits below the mixing height output by the model are considered to be within the ML. (b) MODIS active fire data was gridded and counted for each BT. Ensemble of trajectories pass through 42080 fires.

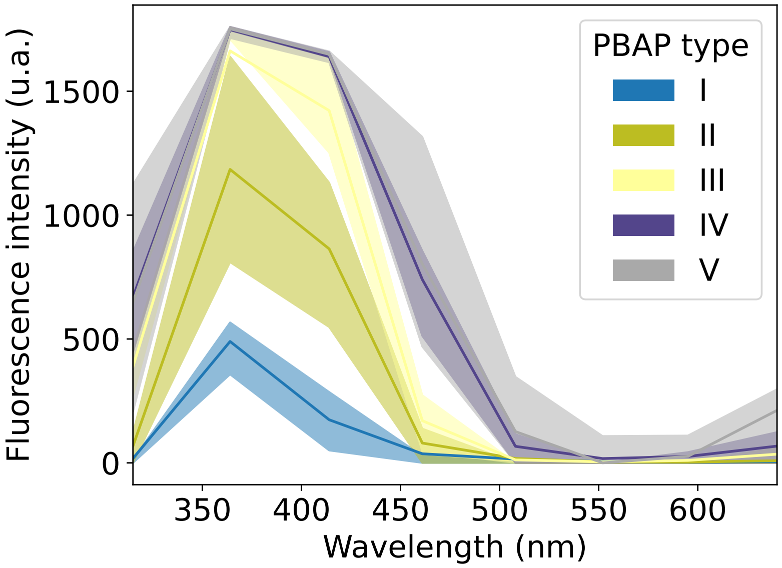


**Figure S7. Spectral comparison of different primary biological aerosol particles (PBAP) types.** Solid line represents median values while shaded area represents the interquartile range.

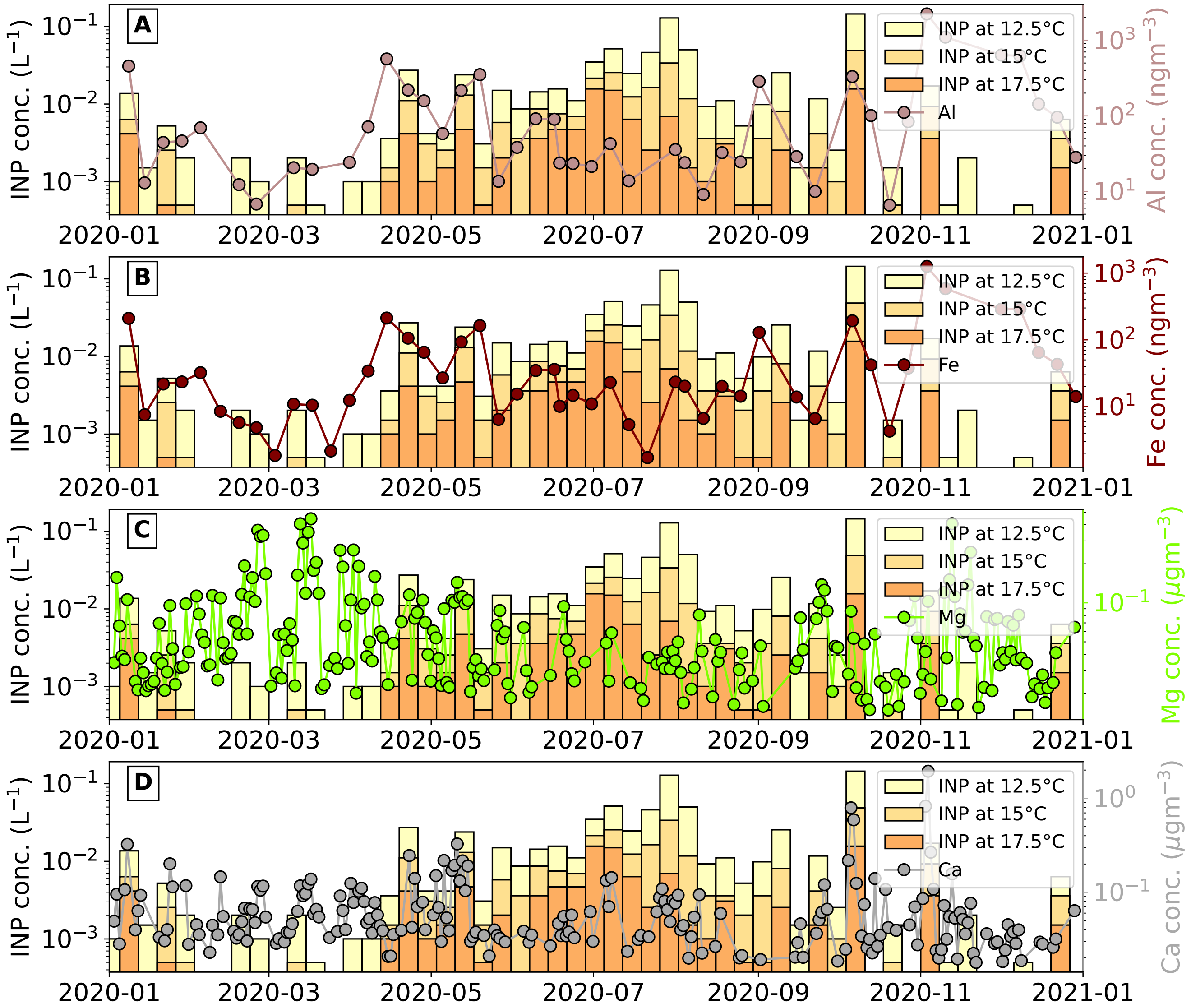


**Figure S8.** **Comparison between dust tracers and ice nucleating particles (INP) concentrations.** Comparison between the yearly trend through 2020 of particulate matter concentrations of aluminum (a, Al), iron (b, Fe), magnesium (c, Mg) and calcium(d, Ca) with INP concentrations at three different high temperatures.

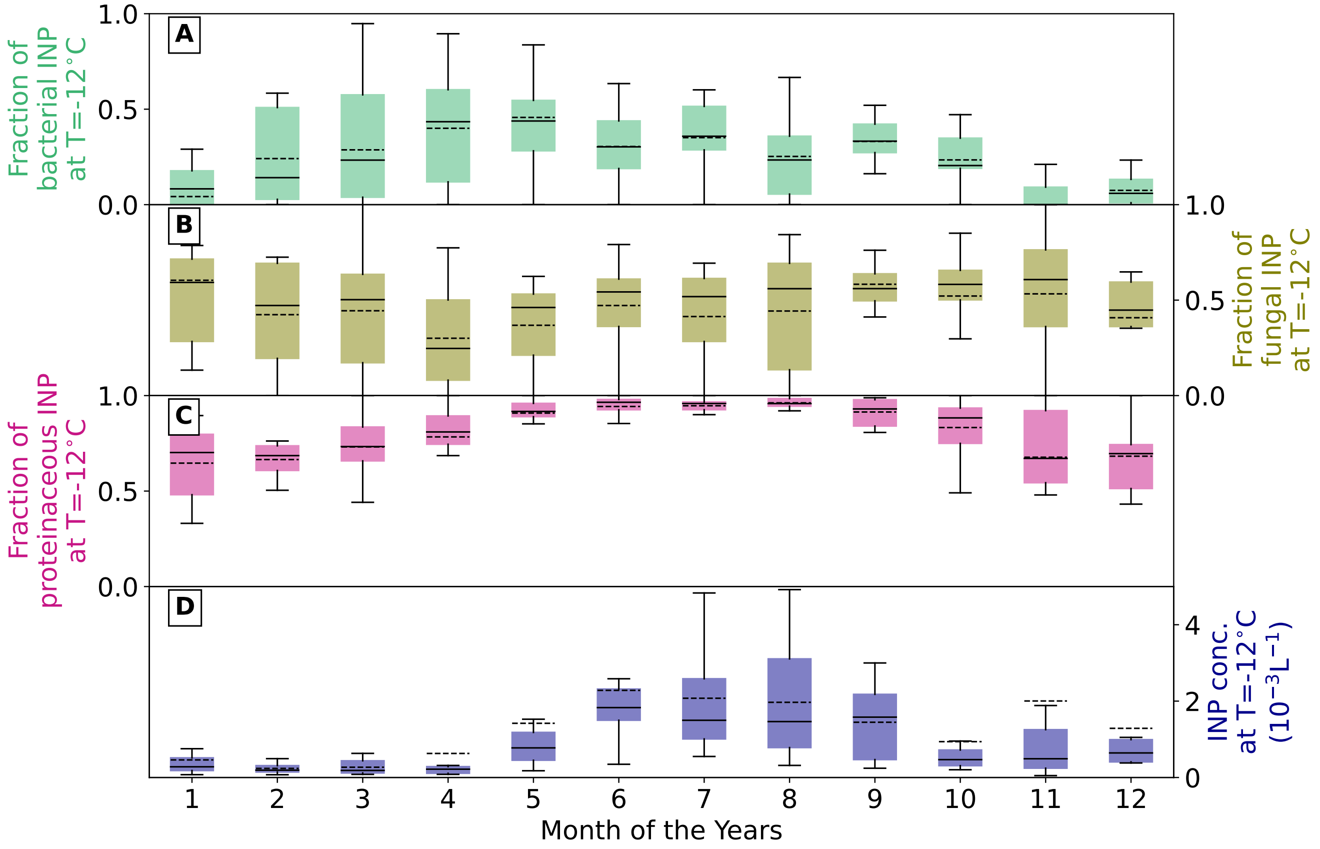


**Figure S9. Annual cycle of bacterial, fungal and proteinaceous fraction of ice nucleating particles (INP) active at temperature T = -12 °C across all four years of measurements.** Boxplots of bacterial (a), fungal (b) and proteinaceous (c, bacterial + fungal) fractions of INP active at temperature T = -12 °C. Total concentration is also shown (d). For the boxplots, the continuous line represents the median and the dashed line the mean, the extent of the colored box shows the interquartile range and the whiskers the data range (1.5 times the interquartile range from the nearest quartile).

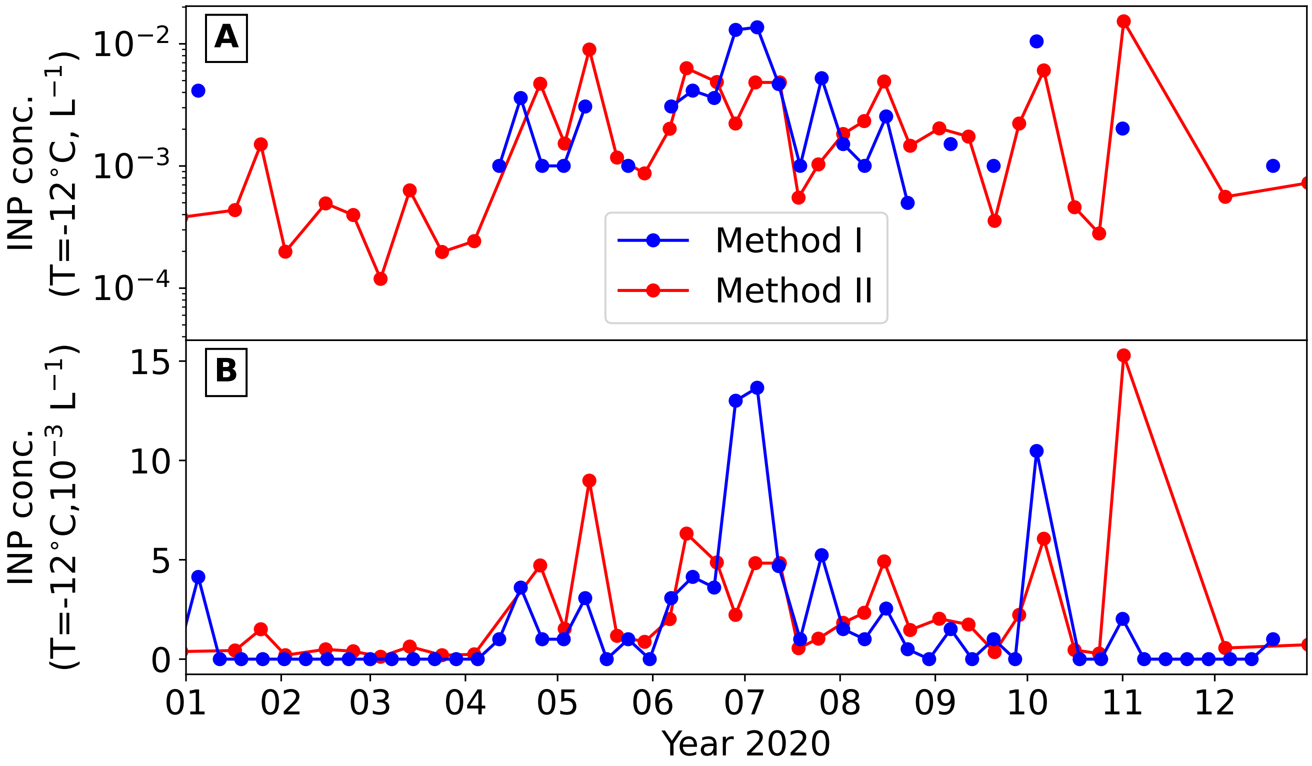


**Figure S10. Comparison between ice nucleating particles (INP) measurement methods I and II for INP active at temperature T = -12 °C.** (a) Using logarithmic scale and (b) linear scale.

# 1 Microscopy analysis of PBAP and comparison to scattering-retrieved morphology parameters

Transmission electron microscopy (TEM) analysis was applied to coarse particle samples (aerodynamical size $> 1\,\mu$m) that were collected simultaneously on the same sampling line as the multiparameter bioaerosol spectrometer (MBS). These samples were collected in December 2019 (1 sample), August 2020 (4 samples), and September 2020 (1 sample, Table S1). Here, we present a case study of the two samples collected on August 7, 2020, with a total of 75-minute sampling period. We chose these two samples to have better statistics for comparison. Since they were taken on the same day, they are likely to have been emitted from the same sources. TEM images of primary biological aerosol particles (PBAP) found in other samples are shown in Figure S13. In total, 13 PBAP were classified according to the TEM composition analysis[1](Figures 1e-h; Table S1 and Figure S12). PBAP identified by TEM analysis represented approximately 4% of all analyzed particles or $\sim 0.2\,L^{-1}$, which is comparable to the MBS derived PBAP concentration of $0.11\,L^{-1}$ for the same day. An example of the elemental analysis of the particle in panel (e) of Figure 1 in the main manuscript is shown in Figure S11 (panels a-d). Carbon (a) is concentrated mainly in the hemispheres of the PBAP, while potassium (b) and phosphorus (c) are concentrated in small inclusions. Nitrogen is seen throughout the particle (d). Some particles had structures that resembled pili and flagellum (such as in the lower right corner of panel G in Figure S12). Most PBAP particles had the same slightly ellipsoidal shape, while one particle had a long tail and another was found to be attached to an organic particle (Figures 1-f and g in the main manuscript, respectively). Their equivalent area diameters ranged from 1.5 to 4 $\mu$ m, with a diameter mode between 2-3 $\mu$m, which compared well with the optical diameter range (1-7 $\mu$m) and mode (2-4 $\mu$m) of PBAP measured by the MBS (see panel e in Figure S11), demonstrating that both methods independently derived similar sizes. PBAP were probably mostly represented by bacteria or fungal spores according to their shape, composition, structure, size and presence of flagellum[2]. Arctic INP (T = -20 °C) have been observed in summer to be dominated by particles in the size range 1.2-3 $\mu$m, deviating from the rest of the year when larger particles are dominating (3-12 $\mu$m, see supplementary Figure 15 of Creamean *et al.* 2022[3]). Our findings seem to reflect these results.

During the same day, the MBS measured 48 PBAP particles, representing 0.07% of the CP. Their contribution by type per size is shown in Figure S11-f, revealing the presence of the five PBAP types identified by the MBS during the coarse of this day. Furthermore, the mean scattering signal (and interquartile) in both linear detector arrays of the MBS is shown in Figure S11-g as an average of all detected PBAP. This scattering signal resembles that of symmetric particles (mean asymmetry parameter of 24, i.e. the degree of dissimilarity between both linear detector signals on a scale from 1 to 100) and slightly elongated particles (peak-to-mean ratio of 3.05, i.e. the ratio between the most intense pixel and the mean value across each individual array)[4]. Thus, the scattering signal measured by the MBS for the PBAP represented the morphology (symmetrical and slightly ellipsoidal) observed in the TEM images.

**Table S1.** Classification of coarse mode (diameter $> 1\,\mu$m) particles analysed by transmission electron microscopy per sample.

| Sample | Biological | Dust | Sea salt | Aluminum | Sulfate | Carbonaceous | Others | Total |
|---|---|---|---|---|---|---|---|---|
| 2019-12-10 | 0 | 6 | 86 | 16 | 6 | 1 | 3 | 118 |
| 2020-08-07 (1) | 8 | 2 | 74 | 0 | 32 | 4 | 1 | 121 |
| 2020-08-07 (2) | 5 | 0 | 118 | 0 | 32 | 12 | 7 | 174 |
| 2020-08-09 | 4 | 3 | 122 | 0 | 2 | 0 | 0 | 131 |
| 2020-08-26 | 2 | 1 | 30 | 0 | 13 | 3 | 4 | 53 |
| 2020-09-06 | 0 | 0 | 116 | 0 | 2 | 1 | 4 | 123 |

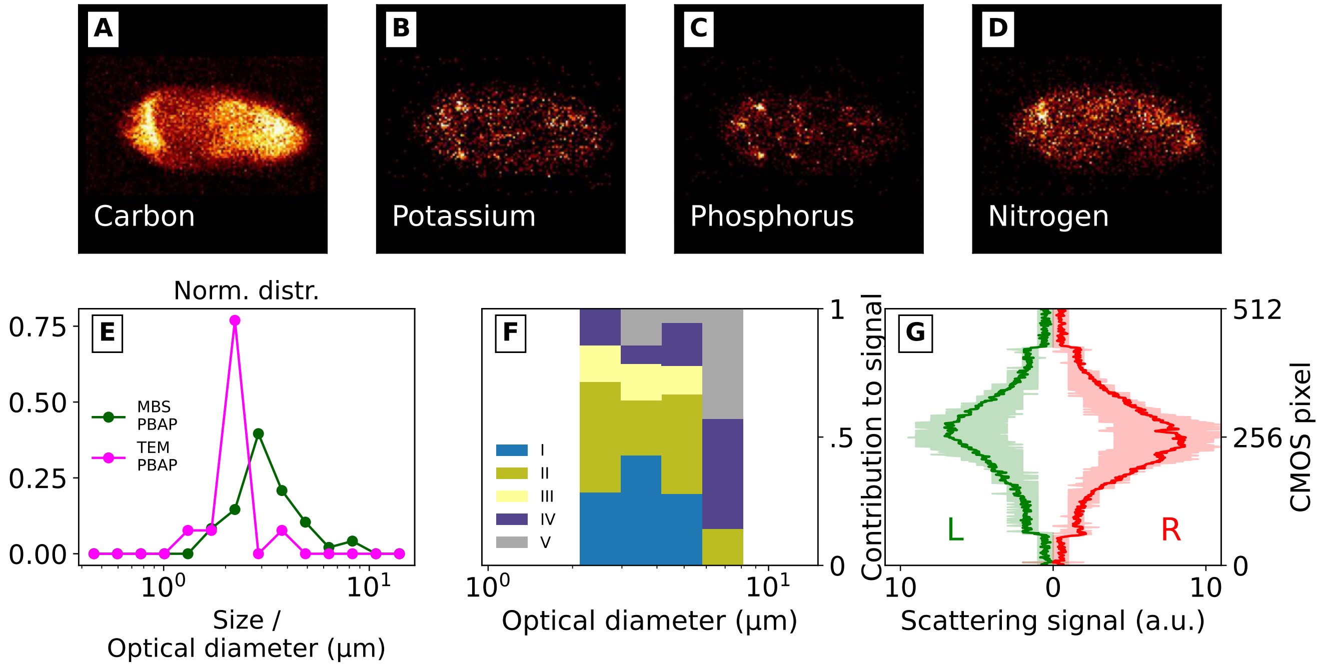


**Figure S11. Transmission electronic microscopy (TEM) images and multiparameter bioaerosol spectrometer (MBS) signal from the 7th of August of 2020.** (a-d) Composition analysis of the primary biological aerosol particles (PBAP) shown at panel (e) of Figure 1 in the main manuscript. e) Normalized size distribution of PBAP measured by TEM and of PBAP measured by the MBS. f) Spectral signature contribution at each size, measured by the MBS. g) Mean and interquartile scattering signal (for the left and right detectors, L and R respectively) of PBAP particles by the MBS during the day.

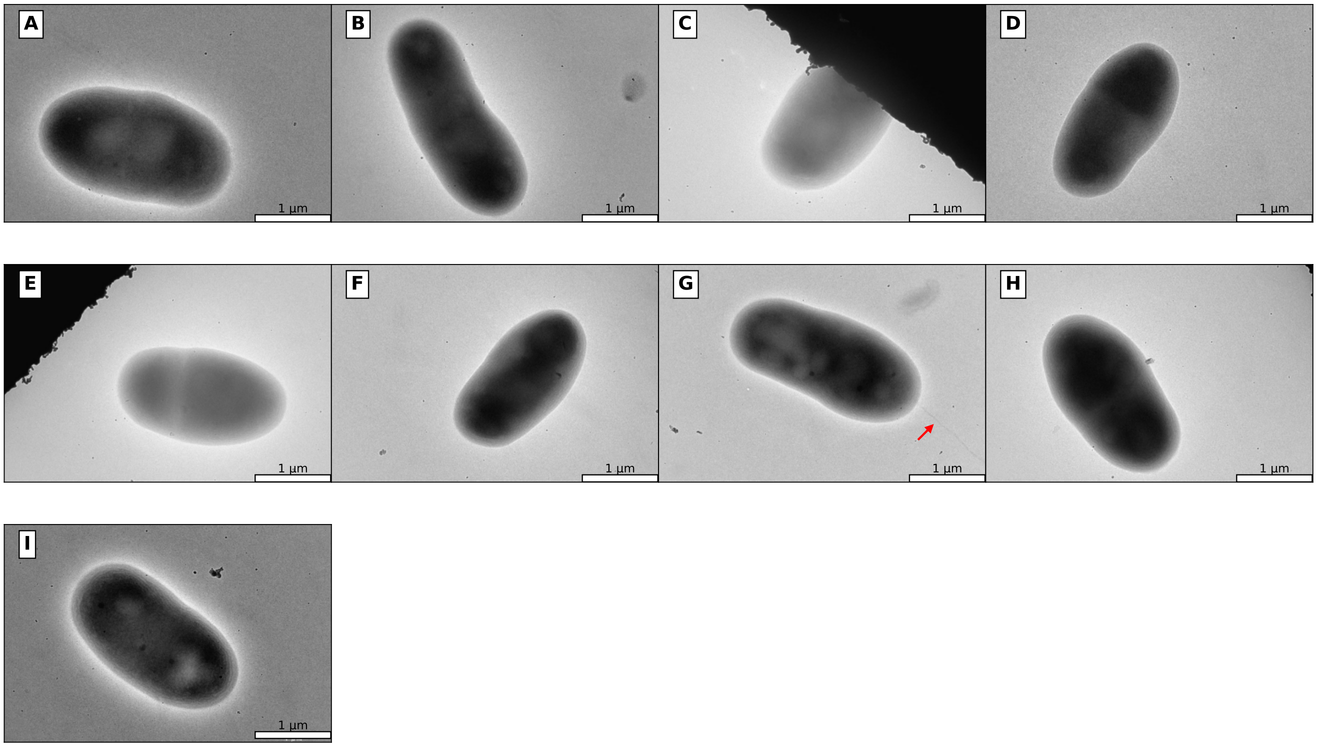


**Figure S12. Transmission electronic microscopy (TEM) images of bioaerosols.** (a-i) Different primary biological particles measured by TEM within two samples collected on the 7th of August of 2020. Red arrow in panel (g) points to a possible flagellum.

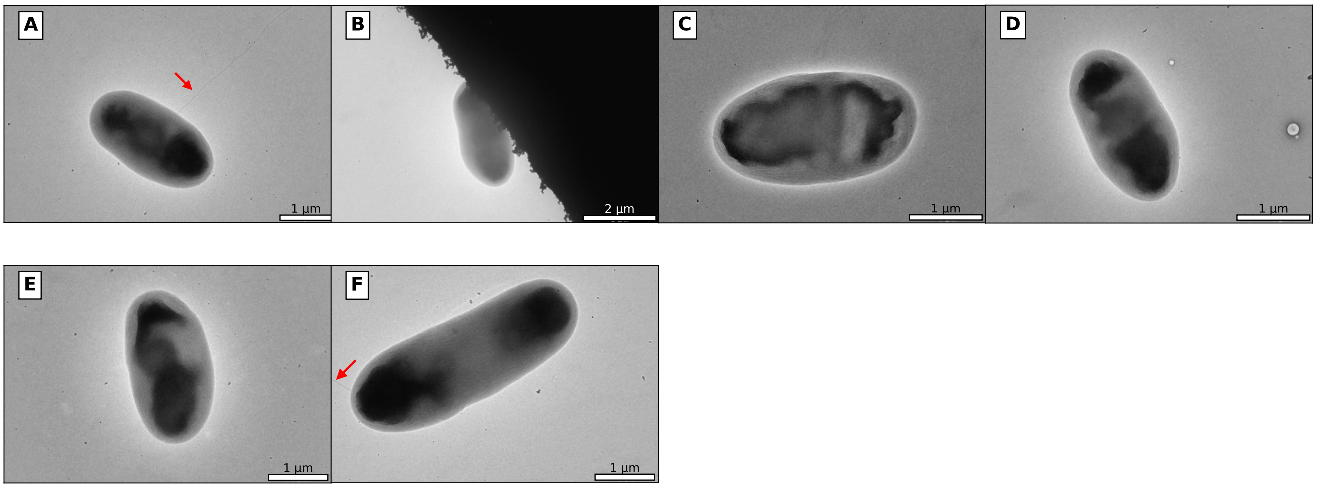


**Figure S13.** **Transmission electronic microscopy (TEM) images of bioaerosols.** Different bioaerosols measured by TEM collected on the 9th of August (a-d) and 26th of August (e,f). Red arrows point to possible flagellum in the images.

## Supplementary References


**1.** Adachi, K. *et al.* Mixing states of Amazon basin aerosol particles transported over long distances using transmission electron microscopy. *Atmospheric Chemistry and Physics* **20**, 11923–11939 (2020).

**2.** Pósfai, M., Li, J., Anderson, J. R. & Buseck, P. R. Aerosol bacteria over the Southern Ocean during ACE-1. *Atmospheric Research* **66**, 231–240 (2003).

**3.** Creamean, J. M. *et al.* Annual cycle observations of aerosols capable of ice formation in central Arctic clouds. *Nature Communications* **13**, 1–12 (2022).

**4.** Crawford, I. *et al.* Detection of airborne biological particles in indoor air using a real-time advanced morphological parameter uv-lif spectrometer and gradient boosting ensemble decision tree classifiers. *Atmosphere* **11** (2020).
